# Clustering of gene expression data: performance and similarity analysis

**DOI:** 10.1186/1471-2105-7-S4-S19

**Published:** 2006-12-12

**Authors:** Longde Yin, Chun-Hsi Huang, Jun Ni

**Affiliations:** 1 Department of Computer Science & Engineering, University of Connecticut, Storrs, CT 06269, USA; 2 Department of Computer Science, the University of Iowa, Iowa City, IA 52242, USA

## Abstract

**Background:**

DNA Microarray technology is an innovative methodology in experimental molecular biology, which has produced huge amounts of valuable data in the profile of gene expression. Many clustering algorithms have been proposed to analyze gene expression data, but little guidance is available to help choose among them. The evaluation of feasible and applicable clustering algorithms is becoming an important issue in today's bioinformatics research.

**Results:**

In this paper we first experimentally study three major clustering algorithms: Hierarchical Clustering (HC), Self-Organizing Map (SOM), and Self Organizing Tree Algorithm (SOTA) using Yeast *Saccharomyces cerevisiae *gene expression data, and compare their performance. We then introduce *Cluster Diff*, a new data mining tool, to conduct the similarity analysis of clusters generated by different algorithms. The performance study shows that SOTA is more efficient than SOM while HC is the least efficient. The results of similarity analysis show that when given a target cluster, the *Cluster Diff *can efficiently determine the closest match from a set of clusters. Therefore, it is an effective approach for evaluating different clustering algorithms.

**Conclusion:**

HC methods allow a visual, convenient representation of genes. However, they are neither robust nor efficient. The SOM is more robust against noise. A disadvantage of SOM is that the number of clusters has to be fixed beforehand. The SOTA combines the advantages of both hierarchical and SOM clustering. It allows a visual representation of the clusters and their structure and is not sensitive to noises. The SOTA is also more flexible than the other two clustering methods. By using our data mining tool, *Cluster Diff*, it is possible to analyze the similarity of clusters generated by different algorithms and thereby enable comparisons of different clustering methods.

## Background

Microarray technology is one of the latest breakthroughs in experimental molecular biology. The technology permits the analysis of gene expression, DNA sequence variation, protein levels, tissues, cells and other chemicals in a massive format [1,2]. However, the analysis and handling of such fast growing data is becoming one of the major bottlenecks in the utilization of the technology. Powerful mathematical and statistical methods are therefore called for this purpose to search for orderly features and logical relationships in such data.

Several clustering methods (algorithms) have been proposed for the analysis of gene expression data, such as Hierarchical Clustering (HC) [3], self-organizing maps (SOM) [4], and k-means approaches [5]. Although many of the proposed algorithms have been reported to be successful, no single algorithm has emerged as a method of choice. Further, the issues of determining the "correct" number of clusters and the choice of "best" algorithm are not yet clear [6].

In this paper we first experimentally study three major clustering algorithms: Hierarchical Clustering (HC), Self-Organizing Map (SOM), and Self Organizing Tree Algorithm (SOTA) [7] using Yeast *Saccharomyces cerevisiae *gene expression data and compare their performance. Then, we present a new data mining tool, *Cluster Diff*, which allows the similarity analysis of clusters generated by different algorithms. A case study is conducted based on clusters generated by SOTA and SOM.

## Results and Discussion

### Performance study

We use GEPAS (Gene Expression Pattern Analysis Suite) to conduct our performance study on three major clustering algorithms: Hierarchical Clustering (HC), Self-Organizing Map (SOM), and Self Organizing Tree Algorithm (SOTA) using Yeast *Saccharomyces cerevisiae *gene expression data.

**Figure 1:**
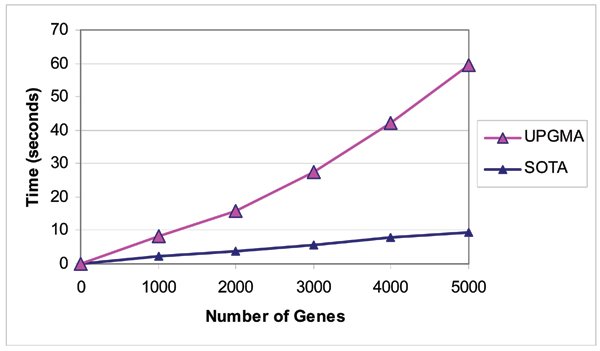
**Runtimes for SOTA and hierarchical**. For a large number of genes (>1000), SOTA is obviously faster than HC. However, for a relatively small number (<1000) of genes, the performance of the SOTA and that of HC method are similar.

The runtime comparison (SOTA vs. HC) results are shown in Figure 1. For a large number of genes (>1000), SOTA is faster than HC. For 5000 genes, it is about three orders of magnitude faster. However, for a relatively small number (<1000) of genes, the performance of the SOTA and HC methods are similar. In fact, for less than 600 genes the computation using the HC method is slightly faster. This is because the training of the neural network implies a minimum number of presentations [8].

**Figure 2:**
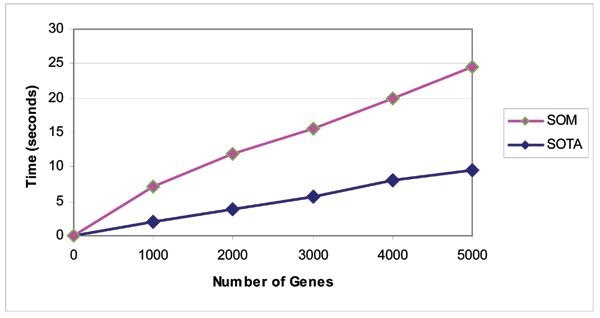
**Runtime for SOM and SOTA**. The runtime of SOTA and SOM are proportional to the sample sizes, and the computation using SOTA is faster than the SOM.

The runtime comparison (SOTA vs. SOM) results are shown in Figure 2. From this figure we know that the runtime of SOTA and SOM are proportional to the sample sizes, and the computation using SOTA is faster than the SOM.

In summary, SOTA is more efficient than SOM while HC is the worst. The SOTA is much faster than HC method. However, this is not always true when the data set is small. The runtimes of SOTA and SOM are approximately proportional to the number of genes. They both can be used to handle very large data sets.

### Clustering results

**Figure 3:**
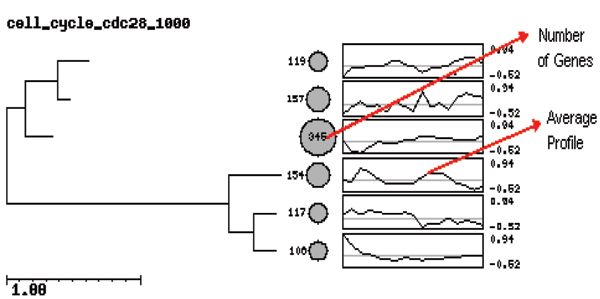
**Clustering result of SOTA**. The size of the ratio of the circles is proportional to the amount of genes in that cluster. The patterns of the clusters appear on the right of the circles.

The result of SOTA clustering is shown in Figure 3. In this plot, the size of the ratio of the circles is proportional to the amount of genes in that cluster. The patterns of the clusters appear on the right of the circles.

**Figure 4:**
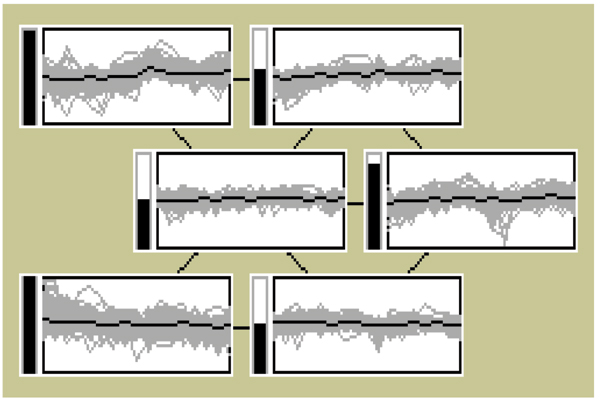
**Clustering results of SOM**. Each rectangle corresponds to a node of the map. The black thick line in the rectangle corresponds to the profile of the node, and the grey lines correspond to the profiles of the genes in that cluster. The black bars on the left of the profiles are proportional to the number of genes in the clusters.

The clustering result of SOM is shown in Figure 4. Each rectangle corresponds to a node of the map. The black thick line in the rectangle corresponds to the profile of the node, and the grey lines correspond to the profiles of the genes in that cluster. The black bars on the left of the profiles are proportional to the number of genes in the clusters.

### Cluster similarity analysis

We analyze the similarity of clusters generated by SOTA and SOM with our data mining tool *Cluster Diff*.

The cluster similarity analysis results (SOTA vs. SOM) are summarized in Table 2. One of the screenshots is shown in Figure 6. The score in bold bears the maximum value in both the row and the column, and the score in italic bears the maximum value in either the row or the column, but not both. From this table, we can find that most SOTA clusters match the SOM clusters well and vice versa. An example of a good match (0.46) is SOTA1 with SOM22 (See Figure 7.). The profiles of these two clusters have similar trends, meaning that most genes in the two clusters are similar.

Two clusters are mismatched if the score is 0.00. An example is SOTA6 with SOM11 (See Figure 8.). From this figure, we can tell that their trends are different. The cluster similarity analysis results can better be viewed by rearranging Table 2 in a similar way as Table 3.

## Conclusion

HC methods allow a visual, convenient representation of genes. They can also generate an order of the genes, though the order is not unique. However, they are neither robust nor efficient. The SOM, as a neural network, is more robust against noise. The effects of outliers can be counter-balanced or corrected by the sequence of input genes. A disadvantage of SOM is that the number of clusters has to be fixed beforehand. But, in practice, that information may not be known. The SOTA is based on both neural networks and HC methods. It combines the advantages of both hierarchical and SOM clustering. It allows a visual representation of the clusters and their structure and is not sensitive to noises. The SOTA is also more flexible than the other two clustering methods.

Performance study shows that SOTA is more efficient than SOM while HC is the worst. The runtimes of SOTA and SOM are approximately proportional to the number of genes. They both can be used to handle very large data sets.

In this paper, we also present a data mining tool, *Cluster Diff*, which allows the similarity analysis of clusters generated by different algorithms. This tool may: (1) improve the quality of the data analysis results, (2) support the prediction of the number of relevant clusters in the microarray datasets, and (3) provide cross-reference between different algorithms. The software tool can also be used to analyze cluster similarities from other biomedical data.

## Methods

### Clustering methods

Clustering methods can be used to determine the natural sub-groups in a data set. They do not need previous knowledge before analysis [9,10]. In this section, we briefly depict three commonly-used clustering methods from the collection of clustering algorithms developed in the past decades [3-5,7,11-18], including the classic Hierarchical Clustering (HC) methods, the Self-Organizing Map (SOM) neural networks, and the Self-Organizing Tree Algorithm (SOTA).

### Hierarchical Clustering (HC)

HC methods are useful for analyzing gene expression data as well as many data in other contexts. They are agglomerative (bottom-up) approaches [3]. The clustering process starts with each gene as an individual cluster. These clusters are then successively merged together to form new, larger clusters until all of the genes are in one big cluster. The sequence of clusters is represented by a hierarchical binary tree, the *dendogram *[8], which can be cut at a specific hierarchical level to obtain a desired number of clusters. The topology of the clusters is a binary tree. During the clustering process, the number of clusters can only be reduced. The HC methods are deterministic, as each gene will be assigned to one and only one cluster. A large number of clusters will be produced, which is a valuable feature for data structure discovery. The clustering process will also produce an order for the genes, and the order is informative for gene display. However, the order of genes is not unique because the two branches of each cluster can be switched without any problem. These methods also have some disadvantages. For example, the optimal merge of two clusters at each step may lead to a sub-optimal cluster hierarchy overall. Because of the deterministic characteristics of the HC methods, a bad assignment made earlier cannot be corrected.

### Self-Organizing Map (SOM) neural network

SOM [14] is a neural network with a number of nodes or neurons. Usually the configuration of these nodes is rectangular or hexagonal [15,19]. The nodes have an associated vector of the same length of the input data. All nodes have initial random values and the reference vectors are adjusted during the training process. After the network is stable, these reference vectors are used to group the genes based on the closeness of the genes to the reference vectors.

During the training stage, the strength of the updating of the reference vectors depends on their distances to the winner vector, which is the closest vector to a randomly selected gene. The training length, the training rate, and the size of the updating neighborhood can be customized. Usually the training is performed in two phases: the first one is the ordering phase (strong training rate and large updating radius) and the last one is the fine-tuning phase (long training length with a weak training rate and a smaller radius).

The SOM clustering method is non-deterministic, owing to the random order in which genes are used to move the reference vectors. It is not sensitive to gene outliers (noises), because the effects of outliers can be counter-balanced or corrected through the input of other genes. Once the configuration for partitions of the decision space is chosen, the number of clusters is determined and is fixed during the rest of clustering process. The k-means clustering methods also have a fixed, pre-determined number of clusters at the beginning. However, the Self-Organizing Map method is different in that the cluster centres are restricted to lie in a one or two-dimensional manifold (the decision space).

### Self-Organizing Tree Algorithm (SOTA)

Contrary to the HC methods, which are agglomerative clustering methods, the Self-Organizing Tree Algorithm is a divisive (top-down) clustering method [7,16,20]. It starts the clustering process with a binary tree consisting of a root node with two leaves, each of which represents one cluster. The self-organizing process then grows the tree by converting the leaf with the largest resources into a node and attaching two new leaves to it. The resource value for each cluster is defined as the mean value of the distances between the cluster and the genes associated with it.

The Self-Organizing Tree Algorithm combines the tree structure of hierarchy clustering methods and the neural network structure of Self-Organizing Maps for adjusting the cluster vectors. Similar to the SOM algorithm, the SOTA [7] algorithm is non-deterministic and not sensitive to gene outliers (noises). The topology of the clusters is a binary tree, which is similar to that of the hierarchical algorithm except that the number of clusters can only grow. Furthermore, the number of clusters can be customized using the SOTA method by stopping the self-organizing tree growth process after a specific number of loops. Therefore, the SOTA algorithm is more flexible than the HC method and SOM.

### Performance study

For gene expression data analysis, we should consider the size of the dataset and the noise contained in the data. Both SOM and SOTA are based on neural networks, so they are more efficient than the hierarchical method (algebraic method) in dealing with large amounts of noisy data.

It is claimed in [12] that the SOTA has approximately linear runtime and is much faster than SOM and the Hierarchical methods.

The purpose of our study is to test the performance of the three clustering methods as well as to further compare the results of the SOM and SOTA clustering analysis.

#### Software for performance study

The software tool we use for experimental study is *GEPS (Gene Expression Pattern Analysis Suite) *[8]: It includes the following servers: (a) *Cluster Server*: This is an interface to HC. The resulting dendogram is plotted with TreeView; (b) *SOM Server*: This is an interface to SOM package. The map is plotted with SomPlot. The resulting clusters can be extracted to continue with the analysis; (c) *Sotarray Server*: This is the interface to SOTA for DNA array. The resulting tree can be viewed with TreeView or with SotaTree. The resulting clusters can be extracted to continue with the analysis; (d) *SomTree Server*: This tool combines SOM and HC. The nodes of the resulting SOM map are clustered and the tree is plotted with SotaTree. The resulting clusters can be extracted to continue with the analysis.

#### The data set and data pre-processing

We experiment with a subset of the Yeast *Saccharomycs cerevisiae *data set that measures the expression level of each of the 6601 different genes of *Saccharomycs cerevisia *[21,22]. The data is obtained using an Affymetrix hybridization array and the values in the subset we select are measured at 17 time points sampled at every 10 minutes during approximately two cell division cycles [23].

Our first processing step is to prune genes with more than one missing value. After we complete this process, 5509 genes remained in our data set. The second step is to randomly select 1000, 2000, 3000, 4000, and 5000 genes from the 5509 genes and save them in plain text files. These five pre-processed data sets are used for comparing the algorithms.

### Runtime comparison

#### SOTA vs. HC

UPGMA is an agglomerative HC method. It starts by calculating the all-to-all distance matrix. The two closest patterns are merged and the all-to-all distance matrix is calculated again but using the new cluster instead of the two merged patterns. This process is repeated until the complete dendrogram is built.

Test condition for UPGMA: (a) Cluster method: pairwise arithmetic average; (b) Distance function: correlation coefficient

Test condition for SOTA: (a) Cluster method: pairwise arithmetic average; (b) Distance function: correlation coefficient; (c) Variability threshold: 90%

#### SOTA vs. SOM

Test condition for SOM: (a) Topology: Hexagonal lattices; (b) X-Dimension: 2; (c) Y-Dimension: 3.

Test condition for SOTA: (a) Cluster method: pairwise arithmetic average; (b) Distance function: correlation coefficient; (c) Variability Threshold (%): 90.

### Clustering results

The pre-processed data file with 1 k genes is used to compare the clustering results.

Test condition for Self Organizing Map (SOM): 2 × 3 hexagonal lattices (This will result in 6 clusters).

Test condition for Self Organizing Tree Algorithm (SOTA): (a) Cluster method: pairwise arithmetic average; (b) Distance function: correlation coefficient; (c) Unconditional training stops after 5 cycles (It will result in 6 clusters.).

### Cluster similarity analysis

Many clustering algorithms have been proposed for the analysis of gene expression data, but little guidance is available to help choose among them [24]. For example, they lack facilities for estimating the optimal number of clusters, as well as components for evaluating the quality of the clusters obtained. In this section, we present a software tool that offers similarity analysis of clusters from DNA microarray data.

#### The software

We present a data mining tool, *Cluster Diff*, which allows the similarity analysis of clusters generated by different algorithms. This tool may: (a) improve the quality of the data analysis results, (b) support the prediction of the number of relevant clusters in the microarray datasets, and (c) provide cross-reference between different algorithms. The software tool can also be used to analyze cluster similarities from other biomedical data.

**Figure 5:**
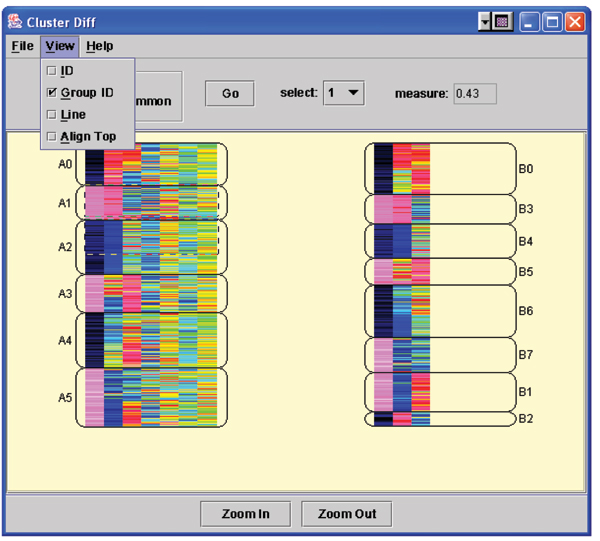
**Screenshot of the *Cluster Diff *window**. The main window contains the file, view, and help buttons. In this figure, the left group (A) has 6 clusters, from A0 to A5; the right group (B) has 8 clusters, from B0 to B7. In each cluster, the column represents the dimension of the Microarray data and the row represents the gene's profile. The score is the measurement of similarity.

The software allows working with two datasets each time. The Main Window (panel, Figure 5) contains the file, view, and help buttons.

In Figure 5, the left group (A) has 6 clusters, from A0 to A5; the right group (B) has 8 clusters, from B0 to B7.

In each cluster, the column represents the dimension of the Microarray data and the row represents the gene's profile. For example, in Figure 5, the group A has 7 dimensions; the group B has 3 dimensions. The score is the measurement of similarity.

**Figure 6:**
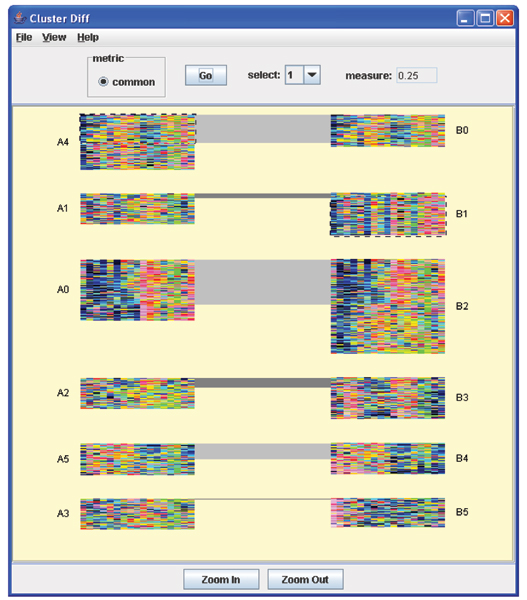
**Screenshot of cluster similarity analysis**. The similarity analysis results of clusters generated by SOTA and SOM. The matched parts are linked by lines in grey colour.

**Figure 7:**
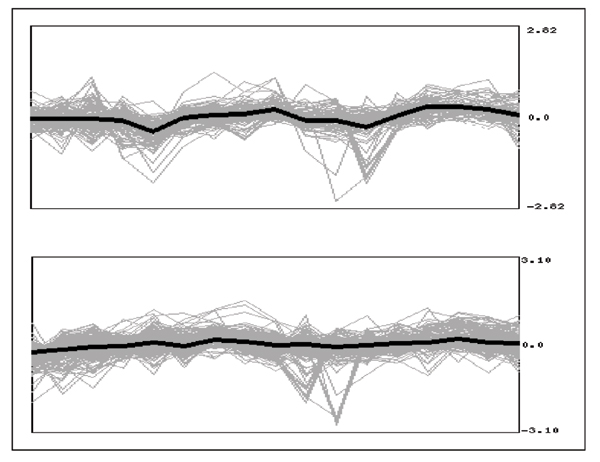
**Example of a good matched clusters**. The profiles of these two clusters have similar trends, meaning that most genes in the two clusters are similar.

**Figure 8:**
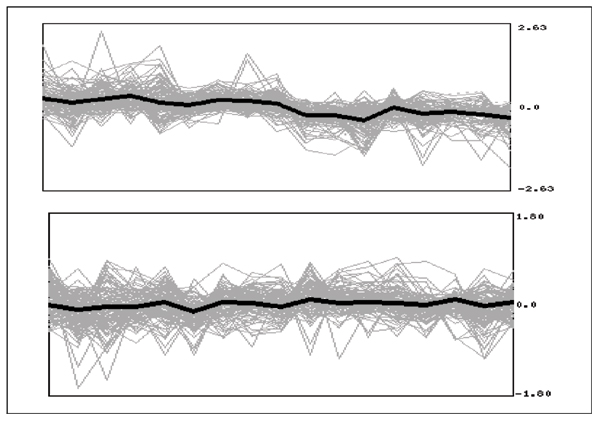
**Example of a bad matched clusters**. Two clusters are mismatched, their trends are different.

The output has multiple visualizations. From button View, you may check different options to get different views. For example, by checking Group ID and Line, the matched parts are linked by lines in gray color (See Figure 6.).

#### Data source and data pre-processing

This tool uses the textual tab-delimited data files. The format is similar to the Stanford tab-delimited format () except that tab [cluster] and [/cluster] should be put between a cluster dataset. An example is shown in Table 1.

#### Cluster similarity analysis method

The pre-processed data files with 1 k genes, after formatting as in Section 4.2, were loaded to the *Cluster Diff *for the cluster similarity analysis.

Each time, we input a pair of clusters, one by SOTA and one by SOM. One of the screenshots is shown in Figure 6. The results are summarized in Table 2

## Authors' contributions

LDY conducted the research and drafted the manuscript. CHH supervised the research and revised the manuscript. JN provided advice and offered feedback throughout this research. All authors read and approved the final manuscript.

## Figures and Tables

**Table 1:** Input data file format.

[cluster]			
YCR008W	-0.26	0.22	-0.2
YDR067C	-0.13	0.15	0.13
YBR211C	-0.53	-0.18	-0.33
[/cluster]			
[cluster]			
YDL228C	-0.83	-0.31	-0.08
YAR075W	-0.68	-0.19	0.64
YBL059W	-0.23	0.04	0.21
[/cluster]			

**Table 2:** Cluster similarity analysis results (SOTA vs. SOM).

	SOM11	SOM12	SOM13	SOM21	SOM22	SOM23	Match
SOTA1	0.00	0.00	0.00	0.04	**0.46**	0.04	SOM22
SOTA2	0.13	0.06	0.00	**0.23**	0.15	0.00	SOM21
SOTA3	**0.40**	*0.15*	0.00	0.16	0.10	0.02	SOM11
SOTA4	0.04	0.14	*0.25*	0.00	0.01	0.13	SOM13
SOTA5	0.00	0.00	0.20	0.00	0.01	**0.32**	SOM23
SOTA6	0.00	0.01	**0.38**	0.00	0.00	0.06	SOM13

Match	SOTA3	SOTA3	SOTA6	SOTA2	SOTA1	SOTA5	

**Table 3:** Rearranged Cluster similarity analysis results (SOTA vs. SOM).

	SOM11	SOM12	SOM13	SOM21	SOM22
SOTA3	**0.40**	*0.15*		0.16	0.10
SOTA4	0.04	0.14	*0.25*		0.01
SOTA6		0.01	**0.38**		
SOTA2	0.13	0.06		**0.23**	0.15
SOTA1				0.04	**0.46**
